# Supplementary material for: SM3DD with segmented PCA: a comprehensive method for interpreting 3D spatial transcriptomics
Source: NAR Genom Bioinform. 2026 Jan 27;8(1):lqag007. doi: 10.1093/nargab/lqag007 (PMC12838529; doi:10.1093/nargab/lqag007)
Supplement: lqag007_Supplemental_Files [file lqag007_supplemental_files.zip › Supplementary Data/Extended Data Table and Supplementary Figure Legends.pdf]

Extended Data Table and Supplementary Figure Legends

Extended Data Table 1 Mean SM3DD comparison of SARS-Cov-2 vs normal lung.

List of P values, directional negative log10(P) and ratios from t-test comparison of mean SM3DDs for SARS-CoV-2 and normal samples.

Extended Data Table 2 SuperPathways indicating widespread interferon γ signaling.

List of SuperPathways from GeneCard Suite's PathCard database that passed FDR control for a prominent cluster of FROM-transcripts that had statistically shorter mean SM3DDs in SARS-CoV-2 samples, indicated by the dark green box in Figure 2B.

Extended Data Table 3 SuperPathways indicating widespread extracellular matrix (ECM) deposition.

List of SuperPathways from GeneCard Suite's PathCard database that passed FDR control for a prominent cluster of FROM-transcripts that had statistically shorter mean SM3DDs in SARS-CoV-2 samples, indicated by the pink box in Figure 2B.

Extended Data Table 4 SuperPathways indicating pathogen phagocytosis

List of SuperPathways from GeneCard Suite's PathCard database that passed FDR control for a prominent cluster of FROM-transcripts that had statistically shorter mean SM3DDs in SARS-CoV-2 samples, indicated by the light green box in Figure 2B.

Extended Data Table 5 SuperPathways indicating pathogen phagocytosis

List of SuperPathways from GeneCard Suite's PathCard database that passed FDR control for a prominent cluster of TO-transcripts that were statistically shorter mean SM3DDs in SARS-CoV-2 samples, indicated by the light green box in Figure 2B.

Extended Data Table 6 SuperPathways identifying SARS-CoV-2 infection and, predominantly, signaling pathways active during infection.

List of SuperPathways from GeneCard Suite's PathCard database that passed FDR control for a prominent cluster of TO-transcripts that were statistically shorter mean SM3DDs in SARS-CoV-2 samples, indicated by the blue box in Figure 2B.

Extended Data Table 7 SuperPathways indicating that cluster may represent the perturbation of immune targeting of self.

List of SuperPathways from GeneCard Suite's PathCard database, identified without FDR control, for a prominent cluster of TO-transcripts that were statistically shorter mean SM3DDs in SARS-CoV-2 samples, indicated by the black box in Figure 2B.

Extended Data Table 8 SuperPathways indicating that cluster may represent the perturbation of immune targeting of self.

List of SuperPathways from GeneCard Suite's PathCard database, identified without FDR control, for a prominent cluster of FROM-transcripts that were statistically shorter mean SM3DDs in SARS-CoV-2 samples, indicated by the black box in Figure 2B.

Extended Data Table 9 Mean SM3DD comparison between 'more' and 'less' pulmonary fibrosis.

List of P values, directional negative log10(P) and ratios from t-test comparison of mean SM3DDs for 'more' and 'less' pulmonary fibrosis.

Extended Data Table 10 SuperPathway 'unfolded protein response' in regions with more pulmonary fibrosis.

List of SuperPathways from GeneCard Suite's PathCard database, identified without FDR control, for a prominent cluster of TO-transcripts that were statistically shorter mean SM3DDs in 'more' fibrotic regions, indicated by the box in Figure 5.

Extended Data Table 11 SuperPathway 'unfolded protein response' in regions with more pulmonary fibrosis.

List of SuperPathways from GeneCard Suite's PathCard database, identified without FDR control, for a prominent cluster of FROM-transcripts that were statistically shorter mean SM3DDs in 'more' fibrotic regions, indicated by the box in Figure 5.

Supplementary Figure 1 Comparison of transcript distances specifically to MZT2A between normal and SARS-CoV-2 infected lungs. Volcano plot of comparisons of transcript distances specifically to MZT2A. X-axis is distance ratio, where larger values represent shorter distances in SARS-CoV-2 infected lungs. Y-axis is negative log10 (P values).

Supplementary Figure 2 Full resolution of unmarked up heatmap from Figure 2B

Clustered directional negative log10(P values) from comparison of transcript distances between normal and SARS-CoV-2 infected lungs.

Supplementary Figure 3 Full resolution of unmarked up heatmap from Figure 4

Clustered directional negative log10(P values) from comparison of transcript distances between 'more' and 'less' pulmonary fibrosis.
