# Supplementary material for: SM3DD with segmented PCA: a comprehensive method for interpreting 3D spatial transcriptomics
Source: NAR Genom Bioinform. 2026 Jan 27;8(1):lqag007. doi: 10.1093/nargab/lqag007 (PMC12838529; doi:10.1093/nargab/lqag007)
Supplement: lqag007_Supplemental_Files [file lqag007_supplemental_files.zip › Supplementary Data/Extended_Data_Table_and_Supplementary_Figure_Legends.docx]

**Extended Data Table and Supplementary Figure Legends**

**Extended Data Table 1 Mean SM3DD comparison of SARS-Cov-2 vs normal lung.**

List of P values, directional negative log10(P) and ratios from t-test comparison of mean SM3DDs for SARS-CoV-2 and normal samples.

**Extended Data Table 2 SuperPathways indicating widespread interferon γ signaling.**

List of SuperPathways from GeneCard Suite’s PathCard database that passed FDR control for a prominent cluster of FROM-transcripts that had statistically shorter mean SM3DDs in SARS-CoV-2 samples, indicated by the dark green box in Figure 2B.

**Extended Data Table 3 SuperPathways indicating widespread extracellular matrix (ECM) deposition.**

List of SuperPathways from GeneCard Suite’s PathCard database that passed FDR control for a prominent cluster of FROM-transcripts that had statistically shorter mean SM3DDs in SARS-CoV-2 samples, indicated by the pink box in Figure 2B.

**Extended Data Table 4 SuperPathways indicating** **pathogen phagocytosis**

List of SuperPathways from GeneCard Suite’s PathCard database that passed FDR control for a prominent cluster of FROM-transcripts that had statistically shorter mean SM3DDs in SARS-CoV-2 samples, indicated by the light green box in Figure 2B.

**Extended Data Table 5 SuperPathways indicating** **pathogen phagocytosis**

List of SuperPathways from GeneCard Suite’s PathCard database that passed FDR control for a prominent cluster of TO-transcripts that were statistically shorter mean SM3DDs in SARS-CoV-2 samples, indicated by the light green box in Figure 2B.

**Extended Data Table 6 SuperPathways identifying SARS-CoV-2 infection and, predominantly, signaling pathways active during infection.**

List of SuperPathways from GeneCard Suite’s PathCard database that passed FDR control for a prominent cluster of TO-transcripts that were statistically shorter mean SM3DDs in SARS-CoV-2 samples, indicated by the blue box in Figure 2B.

**Extended Data Table 7 SuperPathways indicating that cluster may represent the perturbation of immune targeting of self.**

List of SuperPathways from GeneCard Suite’s PathCard database, identified without FDR control, for a prominent cluster of TO-transcripts that were statistically shorter mean SM3DDs in SARS-CoV-2 samples, indicated by the black box in Figure 2B.

**Extended Data Table 8 SuperPathways indicating that cluster may represent the perturbation of immune targeting of self.**

List of SuperPathways from GeneCard Suite’s PathCard database, identified without FDR control, for a prominent cluster of FROM-transcripts that were statistically shorter mean SM3DDs in SARS-CoV-2 samples, indicated by the black box in Figure 2B.

**Extended Data Table 9 Mean SM3DD comparison between ‘more’ and ‘less’ pulmonary fibrosis.**

List of P values, directional negative log10(P) and ratios from t-test comparison of mean SM3DDs for ‘more’ and ‘less’ pulmonary fibrosis.

**Extended Data Table 10 SuperPathway ‘unfolded protein response’ in regions with more pulmonary fibrosis.**

List of SuperPathways from GeneCard Suite’s PathCard database, identified without FDR control, for a prominent cluster of TO-transcripts that were statistically shorter mean SM3DDs in ‘more’ fibrotic regions, indicated by the box in Figure 4.

**Extended Data Table 11 SuperPathway ‘unfolded protein response’ in regions with more pulmonary fibrosis.**

List of SuperPathways from GeneCard Suite’s PathCard database, identified without FDR control, for a prominent cluster of FROM-transcripts that were statistically shorter mean SM3DDs in ‘more’ fibrotic regions, indicated by the box in Figure 4.

**Supplementary Figure 1 Comparison of transcript distances specifically to MZT2A between normal and SARS-CoV-2 infected lungs.** Volcano plot of comparisons of transcript distances specifically to MZT2A. X-axis is distance ratio, where larger values represent shorter distances in SARS-CoV-2 infected lungs. Y-axis is negative log10 (P values).

**Supplementary Figure 2 Full resolution of unmarked up heatmap from Figure 2B**

Clustered directional negative log10(P values) from comparison of transcript distances between normal and SARS-CoV-2 infected lungs.

**Supplementary Figure 3 Full resolution of unmarked up heatmap from Figure 4**

Clustered directional negative log10(P values) from comparison of transcript distances between ‘more’ and ‘less’ pulmonary fibrosis.
