# Supplementary figures and images for: SM3DD with segmented PCA: a comprehensive method for interpreting 3D spatial transcriptomics
Source: NAR Genom Bioinform. 2026 Jan 27;8(1):lqag007. doi: 10.1093/nargab/lqag007 (PMC12838529; doi:10.1093/nargab/lqag007)

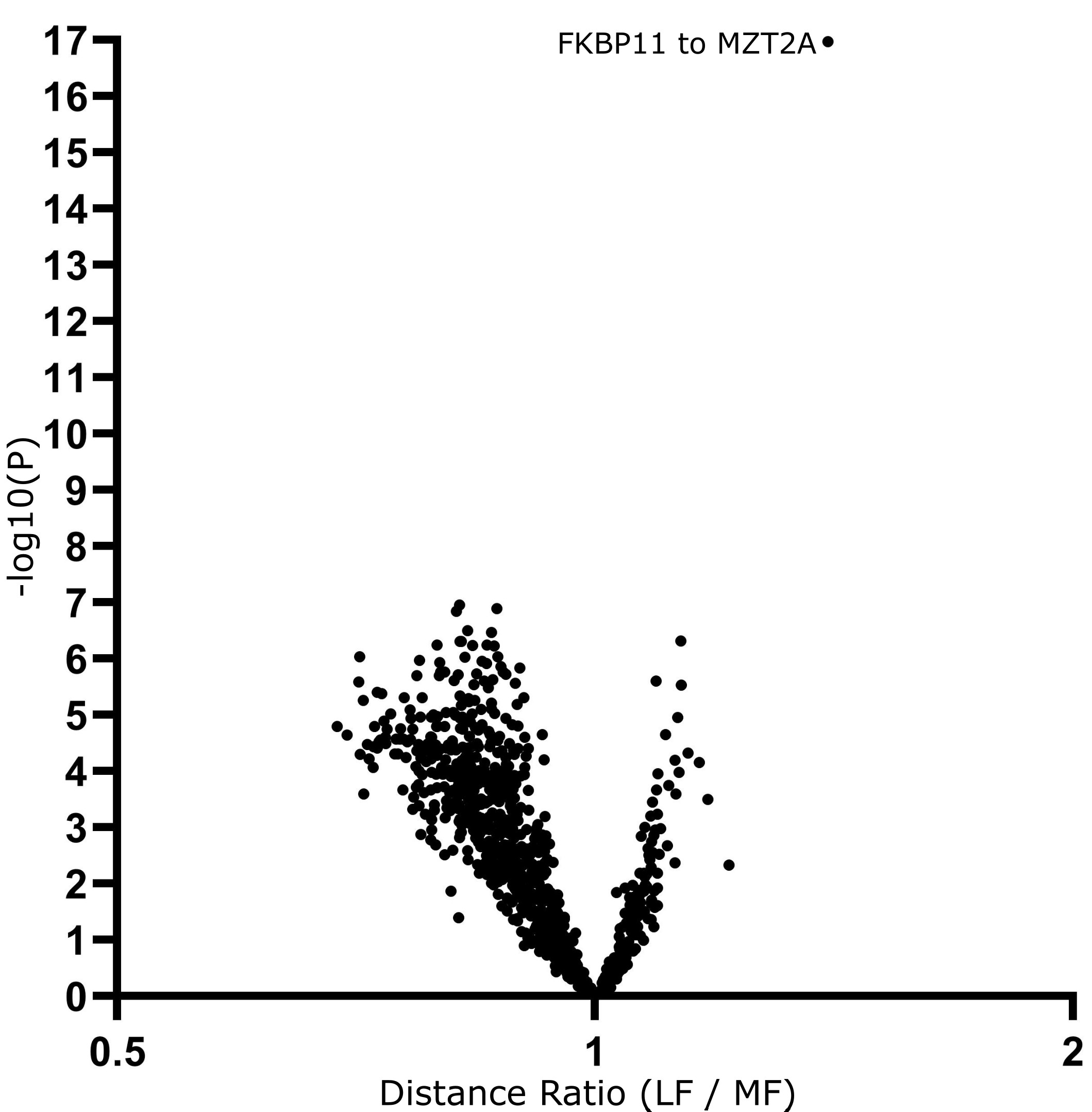

Supplement: lqag007_Supplemental_Files [file lqag007_supplemental_files.zip › Supplementary Data/Supplementary Figure 1.jpg]

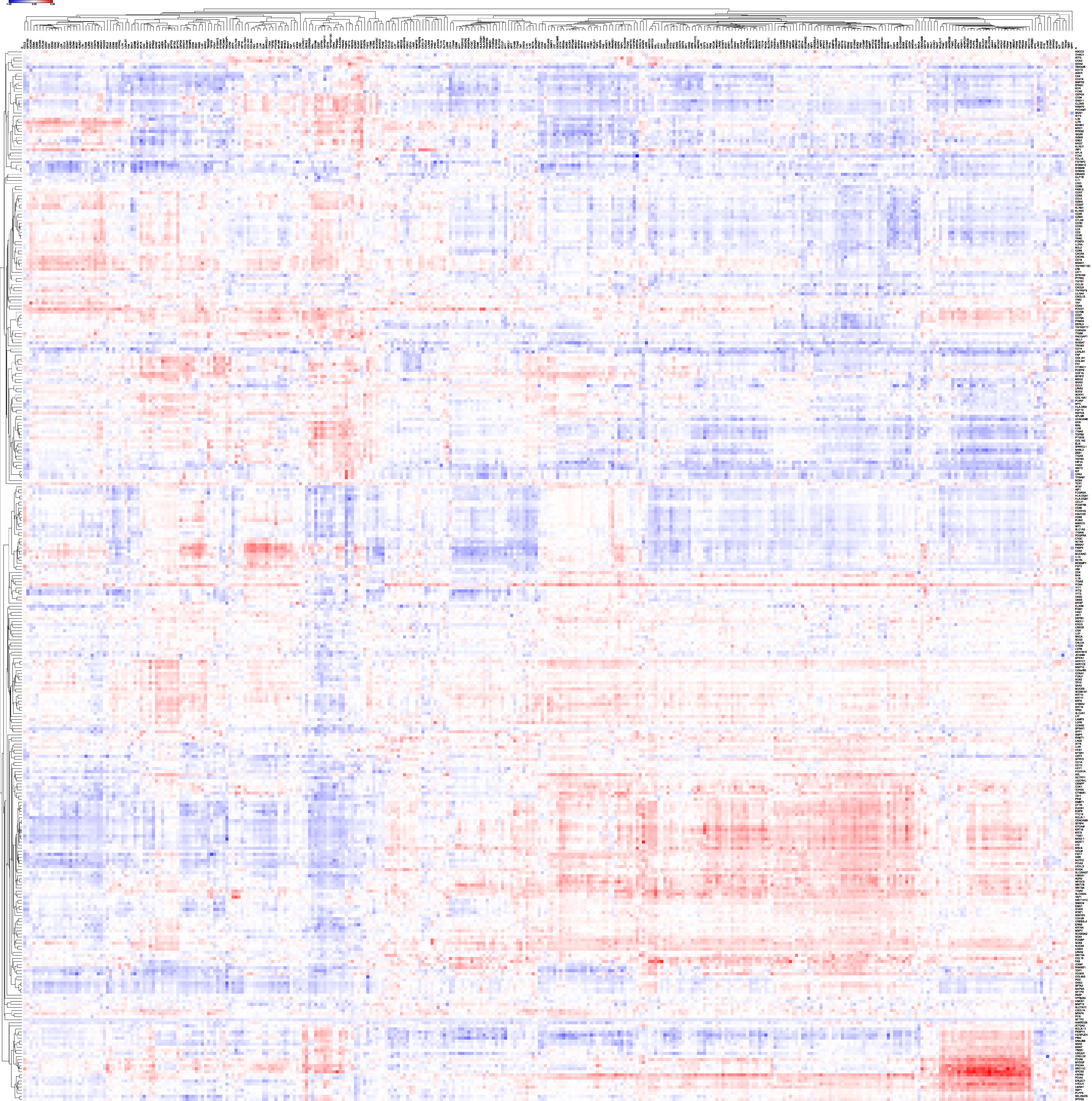

Supplement: lqag007_Supplemental_Files [file lqag007_supplemental_files.zip › Supplementary Data/Supplementary Figure 3.jpg]
